# Intracellular oxygen determined by respiration regulates localization of Ras and prenylated proteins

**DOI:** 10.1038/cddis.2015.64

**Published:** 2015-07-16

**Authors:** A Kim, R Davis, M Higuchi

**Affiliations:** 1 Department of Biochemistry and Molecular Biology, University of Arkansas for Medical Sciences, 4301 West Markham, Little Rock, AR, USA; 2 Department of Urology, University of Arkansas for Medical Sciences, 4301 West Markham, Little Rock, AR, USA

## Abstract

Reduction of mitochondrial DNA (mtDNA) content induces the reduction of oxidative phosphorylation and dependence on fermentative glycolysis, that is, the Warburg effect. In aggressive prostate cancer (PCa), the reduction of mtDNA reduces oxygen consumption, increases intracellular oxygen concentration, and induces constitutive activation of Ras. Many essential proteins for cell death, growth, differentiation, and development, such as Ras, require prenylation for subcellular localization and activation. Prenylation of a protein is defined as the attachment of isoprenoids to a cysteine residue at or near the C-terminus. 3-Hydroxy-3-methyl-glutaryl-coenzyme A reductase (HMGR) produces isoprenoids, and is posttranslationally regulated by oxygen. We investigated a critical role of intracellular oxygen in membrane localization of prenylated proteins. Localization of prenylated proteins (H-Ras, prelamin A/C, and Rab5a) was observed in poorly differentiated PCa (PC-3) and well-differentiated PCa (LNCaP) cells. PC-3 cells exhibited high intracellular oxygen concentration, and H-Ras, prelamin A/C, and Rab5a were localized to various membranes (Golgi and plasma membrane, nuclear membrane, and early endosomes, respectively). Remarkably, exogenous hypoxia (0.2% O_2_) in PC-3 cells induced intracellular hypoxia and changed the localization of the prenylated proteins. H-Ras and Rab5a were translocated to cytosol, and prelamin A/C was in the nucleus forming an abnormal nuclear envelope. The localization was reversed by mevalonate indicating the involvement of mevalonate pathway. In contrast, in LNCaP cells, exhibiting low intracellular oxygen concentration, H-Ras and Rab5a were localized in the cytosol, and prelamin A/C was inside the nucleus forming an inadequate nuclear envelope. Exogenous hyperoxia (40% O_2_) increased the intracellular oxygen concentration and induced Ras translocation from cytosol to the membrane. Prelamin A/C was translocated to the nuclear membrane and formed a proper nuclear envelope. Rab5a was translocated to the early endosomes. The specific localizations of the prenylated proteins were dependent on intracellular oxygen concentration. These results demonstrate that intracellular oxygen concentration regulates the localization and activation of prenylated proteins.

Mitochondrial respiratory function regulates intracellular oxygen concentration.^1^ Reduction of mitochondrial DNA (mtDNA) content induces the reduction of oxidative phosphorylation and dependence on fermentative glycolysis, that is, the Warburg effect.^2, 3^ Reduction of oxidative phosphorylation reduces oxygen consumption, therefore, increases intracellular oxygen concentration. Our previous studies have shown that a reduction of mtDNA induces the aggressive phenotype of prostate cancer (PCa) through increasing oxygen concentration.^4^ The results also showed that the increase in oxygen concentration constitutively activated Ras *via* overexpression of 3-Hydroxy-3-methyl-glutaryl-coenzyme A reductase (HMGR).^4^

Ras is an essential protein of signaling pathways in normal and abnormal cellular functions for cell death, growth, differentiation, and development.^5^ Ras has been the focus of much attention in cancer biology owing to the substantial amount of genetic and/or functional alterations in human cancers.^5^ Ras, a small GTPase, is activated and inactivated by binding to GTP and GDP, respectively.^6^ Ras must localize in the membrane in order to be activated and transduce signals.^5^ The membrane localization of Ras is mediated by prenylation. Many essential proteins, like Ras, require prenylation for subcellular localization and activation. Prenylation of a protein is defined as the attachment of isoprenoids to a cysteine residue at or near the C-terminus.^6^ Most prenylated proteins have a consensus sequence, a CAAX box, at the C-terminus.^7^ Others, like some of Rab family proteins, have C-terminus cysteine residue(s) that serve the same function as the consensus sequence.^7^

Isoprenoids, farnesylpyrophosphate (FPP) and geranylgeranylpyrophosphate (GGPP), are produced in the mevalonate pathway^8^ and regulates prenylation. Prenylated proteins include Ras, nuclear lamins, small GTPases, protein kinases and phosphatase, helicases, and others. The synthesis of FPP and GGPP is regulated by HMGR, a rate-limiting enzyme in the mevalonate pathway.^9^ HMGR synthesizes mevalonate from 3-hydroxy-3-methyl-glutaryl-CoA (HMG-CoA). Hypoxia is known to stimulate the degradation of HMGR.^10^ We hypothesize that intracellular oxygen concentration determined by mitochondria is a critical regulator of localization and activation of prenylated proteins *via* control of prenylation.

## Results

### Determination of H-Ras localization by intracellular oxygen concentration

**Figure 1:**
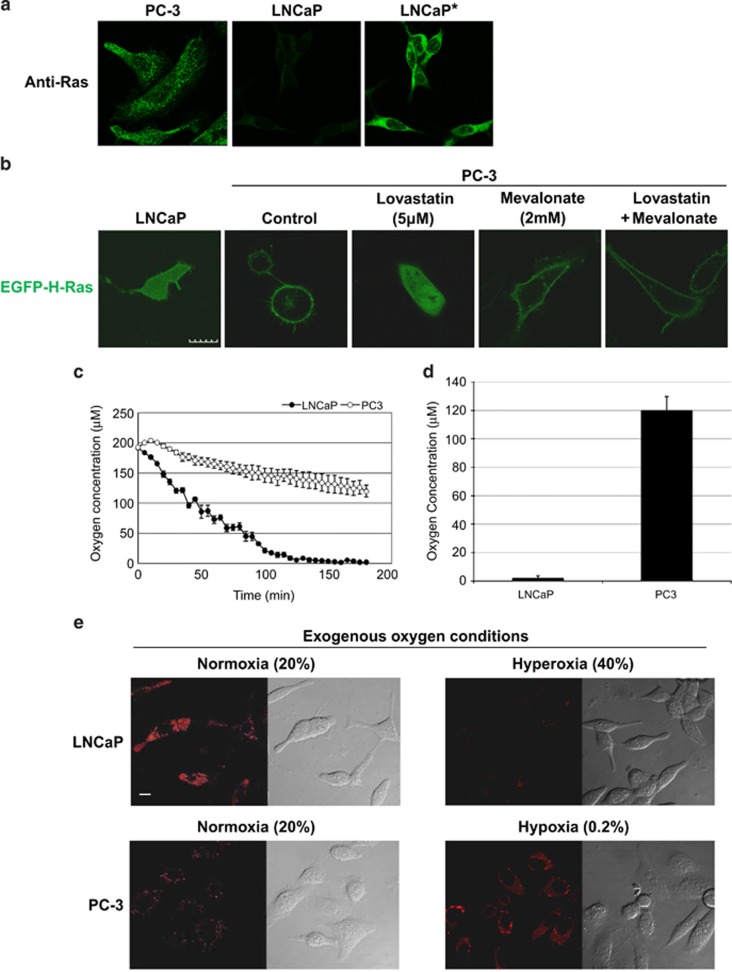
Ras localization is controlled by intracellular oxygen concentration. (**a**) Immunofluorescence analysis of localization of endogenous Ras in LNCaP and PC-3. (* indicates overexposed image of LNCaP). (**b**) Confocal images of EGFP-H-Ras-transfected LNCaP and PC-3 cells. Green fluorescent images show EGFP-H-Ras. Scale bar, 10 *μ*m. EGFP-H-Ras-transfected PC-3 cells were treated with 5 *μ*M lovastatin and/or 2 mM mevalonate. (**c**) and (**d**) oxygen concentration in the medium surrounding the cells. (mean±standard error, *n*=3), **c** is the change in oxygen concentration over time and **d** is the oxygen concentration surrounding the cells at the 3-h time point. (**e**) BTP phosphorescent imaging of the indicated cell lines. LNCaP and PC-3 were cultured at 20% O_2_/5% CO_2_. BTP phosphorescence is indicated by the red images and DIC images show the whole cells. Scale bar, 10 *μ*m

Our previous report demonstrated that well-differentiated PCa cells (LNCaP) had the greater copy number of mtDNA, the higher oxygen consumption, but Ras expression and activation were reduced as compared with those in poorly differentiated PCa cells (PC-3).^4^ Ras is farnesylated on the CAAX-motif located at the C-terminus and is translocated to endomembrane organelles, especially the Golgi apparatus, before arriving at the plasma membrane.^11^ Immunofluorescence analysis demonstrated that in LNCaP, Ras localized only in cytosolic fraction, whereas in PC-3, Ras was present in the endomembrane organelles (Golgi) and plasma membrane^11^ (Figure 1a). Figure 1a also confirmed the previous results that there was significantly less expression of Ras in LNCaP than that in PC-3. To demonstrate the localization of Ras in live cells, LNCaP and PC-3 cells were transiently transfected with a EGFP-H-Ras fusion protein.^11^ In LNCaP cells, cytosolic H-Ras was predominant and was distributed over the entire cell with only a minor amount localized to the plasma membrane (Figure 1b). In contrast, H-Ras in PC-3 cells accumulated in the plasma membrane as well as in the endomembrane organelles (Figure 1b). Ali *et al.*^11^ showed that H-Ras transits the classical secretory pathway from the cytoplasm to the plasma membrane through the Golgi apparatus. A 3-dimensional video of z-stack data from a cell (Supplementary Movie. 1) demonstrated that H-Ras in LNCaP cells primarily localized to the cytosol, whereas in PC-3 cells, H-Ras localized to the plasma membrane and the Golgi (Supplementary Movie. 2). The membrane localization of Ras in PC-3 cells was significantly inhibited by treatment of lovastatin, an inhibitor of HMGR,^12^ and was completely reversed by the addition of mevalonate, a product of HMGR (Figure 1b and Supplementary Movie. 3). Mevalonate treatment alone did not alter the membrane localization of Ras significantly in PC-3 cells (Figure 1b). These data demonstrate that the localization of Ras is regulated by HMGR and is downstream of the mevalonate pathway.

A recent study showed that hypoxia regulates the expression of HMGR *via* stimulating proteasomal degradation.^10^ LNCaP cells showed notably higher oxygen consumption rate than PC-3 cells.^4^ Additionally, the depletion of mtDNA in LNCaP cells led to a reduction of oxygen consumption and an increase in the expression of HMGR, suggesting that mtDNA regulated the oxygen concentration and possibly the expression of HMGR in a cell.^4^ In the current study, the oxygen concentration surrounding cells in an open environment (where atmospheric oxygen freely diffuses into the media) was measured by the Oxoplate. The oxygen concentration in medium surrounding LNCaP cells was significantly less than that surrounding PC-3 cells (Figures 1c and d). To measure the oxygen concentration directly inside a cell, acetylacetonatobis [2-(2'-benzothienyl)pyridinato-k*N*,k*C*^*3*^']iridium(III) (BTP) was used to examine intracellular oxygen concentration in LNCaP and PC-3 cells.^13^ BTP is a hypoxia-sensitive red phosphorescent molecule, which is quenched by available oxygen in a cell.^13^ A significantly higher level of BTP phosphorescence was measured in LNCaP cells compared with PC-3 cells in the atmospheric condition (normoxia) (Figure 1e), clearly demonstrating that LNCaP cells exhibited low intracellular oxygen concentration, whereas PC-3 cells exhibited high intracellular oxygen concentration. LNCaP cells experienced endogenous hypoxia but PC-3 cells did not, possibly because of the significant differences in mitochondrial respiratory activity. LNCaP and PC-3 cells were incubated in exogenous hyperoxic (40% O_2_) and hypoxic (0.2% O_2_) conditions, respectively. Exogenous hyperoxia quenched BTP phosphorescence in LNCaP cells and exogenous hypoxia increased that in PC-3 cells (Figure 1e). These exogenous oxygen conditions were sufficient to regulate the intracellular oxygen concentration in LNCaP and PC-3 cells.

### Regulation of H-Ras localization to membranes *via* various oxygen conditions

**Figure 2:**
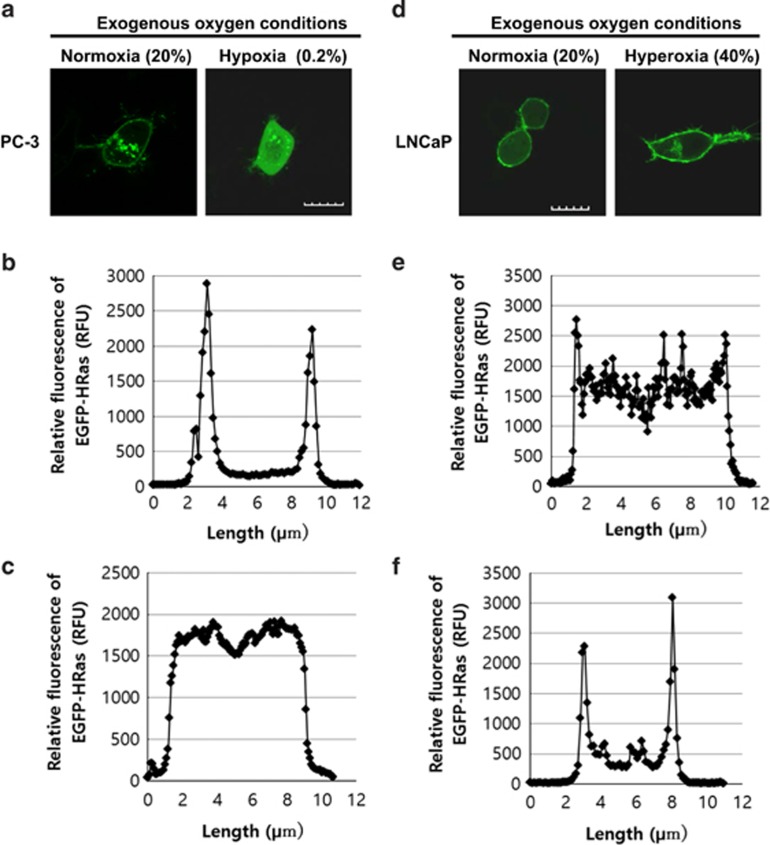
Oxygen concentration determines localization of H-Ras. (**a**) Confocal images of EGFP-H-Ras-transfected PC-3 cells. The cells were treated with indicated oxygen conditions with 5% CO_2_. Scale bar, 10 *μ*m. (**b** and **c**) Each graph illustrates the relative fluorescence values of EGFP-HRas in PC-3 cells throughout a cell. The detailed method to measure the fluorescence values is in Supplementary Figure S1B and S1C. (**d**) Confocal images of EGFP-H-Ras-transfected LNCaP cells. The cells were treated with indicated oxygen conditions with 5% CO_2_. Scale bar, 10 *μ*m. (**e** and **f**) Each graph illustrates the relative fluorescence values of EGFP-HRas in PC-3 cells throughout a cell. The detailed method to measure the fluorescence values is in Supplementary Figure S1B and S1C

**Figure 3:**
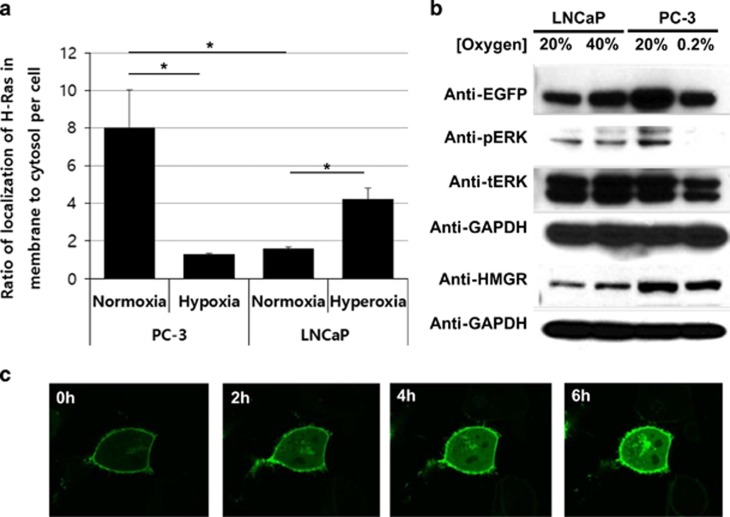
(**a**) The graph illustrates the ratio of localization of H-Ras in membrane to cytosol per cell in the EGFP-H-Ras-transfected LNCaP and PC-3 in the various oxygen conditions, (*n=*7, *P<0.01). The detailed computation is in Supplementary Figure S1B and S1C. (**b**) Western blot for ERK activation and HMGR expression. LNCaP and PC-3 lysates were subjected to SDS-PAGE and analyzed by western blotting. (**c**) Time-dependent changes in localization of H-Ras in LNCaP in exogenous hyperoxic concentration (40% O_2/_5% CO_2_)

Protein prenylation is regulated by the generation of FPP and GGPP.^14^ As HMGR is a rate-limiting enzyme of the mevalonate pathway and is readily degraded by hypoxia,^10^ prenylation of Ras may be regulated by oxygen concentration in a cell. To examine the effect of changes in intracellular oxygen concentration on Ras trafficking, EGFP-H-Ras-transfected LNCaP and PC-3 cells were incubated in various exogenous oxygen concentrations (Supplementary Figure S1A). As shown above, H-Ras localized mostly on the plasma membrane and some in the Golgi in PC-3 with normoxic conditions (Figure 2a). In exogenous hypoxic conditions, the majority of H-Ras in PC-3 cells was translocated to the cytosol (Figure 2a). To further analyze the results, the relative fluorescence of EGFP-H-Ras across a cell was measured by following the method described in supplementary figures and figure legends for Supplementary Figures S1B and S1C. Then, the localizations of the protein in the various oxygen conditions were compared. The relative fluorescence of EGFP-H-Ras in PC-3 cells was notably higher in the membrane than that in the cytosol (Figures 2b and 3a). The exposure to the exogenous hypoxia caused the PC-3 cells to significantly lose the specific membrane localization of the protein, therefore, the relative fluorescence of EGFP-H-Ras in the cytosol was high, and the ratio of membrane to cytosol localization of H-Ras was significantly reduced (Figures 2c and 3a). The 3-dimensional video analysis clearly demonstrated the cytosolic localization of H-Ras in the exogenous hypoxic conditions (Supplementary Movie 4), indicating that the cytosolic localization of H-Ras in exogenous hypoxia was similar to that in the lovastatin-treated PC-3 cells (Supplementary Movies 4 and 3). In LNCaP cells, the majority of H-Ras localized in the cytosol (Figure 2d). The exogenous hyperoxic conditions induced membrane localization of H-Ras in the LNCaP cells (Figure 2d). The relative fluorescence of EGFP-H-Ras in the LNCaP cells was evenly distributed throughout the entire cells and the membrane localization was low (Figures 2e and 3a). The exogenous hyperoxic conditions restored the specific membrane localization of the protein, therefore, the relative fluorescence of EGFP-H-Ras was drastically high in the membrane and the ratio of membrane to cytosolic localization was significantly increased (Figures 2f and 3a). The 3-dimensional video analysis confirmed a decrease in cytosolic H-Ras and an increase in membrane localization of H-Ras to the plasma membrane and Golgi (Supplementary Movie 5). Western blot analysis showed that HMGR expression was decreased by the exogenous hypoxic conditions in PC-3 cells (Figure 3b). These results suggest that HMGR is degraded because of the intracellular hypoxia, and this may lead to a decrease in farnesylation of H-Ras. Western blot analysis also showed that in the exogenous hypoxic conditions, there was less phosphorylation of ERK in PC-3 cells, indicating that Ras activation was inhibited by induced intracellular hypoxia. The data might result from the inhibition of Ras farnesylation and its membrane localization. The time-course analysis demonstrated that the translocation of H-Ras to the Golgi and plasma membrane occurred rapidly (Figure 3c) even within 30 min (Supplementary Movie 6), suggesting that the translocation of H-Ras induced by the enhancement of intracellular oxygen concentration was relatively rapid. These data also suggest that cytosolic H-Ras is apt to be prenylated in order to localize in the membranes. The data from the time-course analysis showed that cytosolic Ras levels also seemed to be increased in the time-course analysis (Figure 3c and Supplementary Movie 6), however, western blotting results showed that there was no significant difference in the levels of EGFP-H-Ras under the various oxygen concentrations (Figure 3b). The increase in membrane EGFP-H-Ras may cause green fluorescence to bleed through the membrane to the cytosol. Western blot analysis showed that HMGR expression and phosphorylation of ERK were increased by exogenous hyperoxic conditions in LNCaP cells (Figure 3b). The western blot analysis also demonstrated that the expression of HMGR in LNCaP cells was significantly lower than that in PC-3, verifying that HMGR was degraded by intracellular hypoxia of LNCaP cells without being incubated in the exogenous hypoxic conditions. These results indicate that an increase in the intracellular oxygen concentration inhibits the hypoxia-stimulated degradation of HMGR, thus, leading to an increase in farnesylation of H-Ras *via* inducing FPP in the mevalonate pathway. The increase in the farnesylation and membrane localization of H-Ras results in the activation of H-Ras and downstream effectors, such as ERK. Overall, the results demonstrate that the localization and activation of H-Ras are critically regulated by intracellular oxygen concentration.

### Regulation of nuclear localization of prelamin A/C by intracellular oxygen concentration

**Figure 4:**
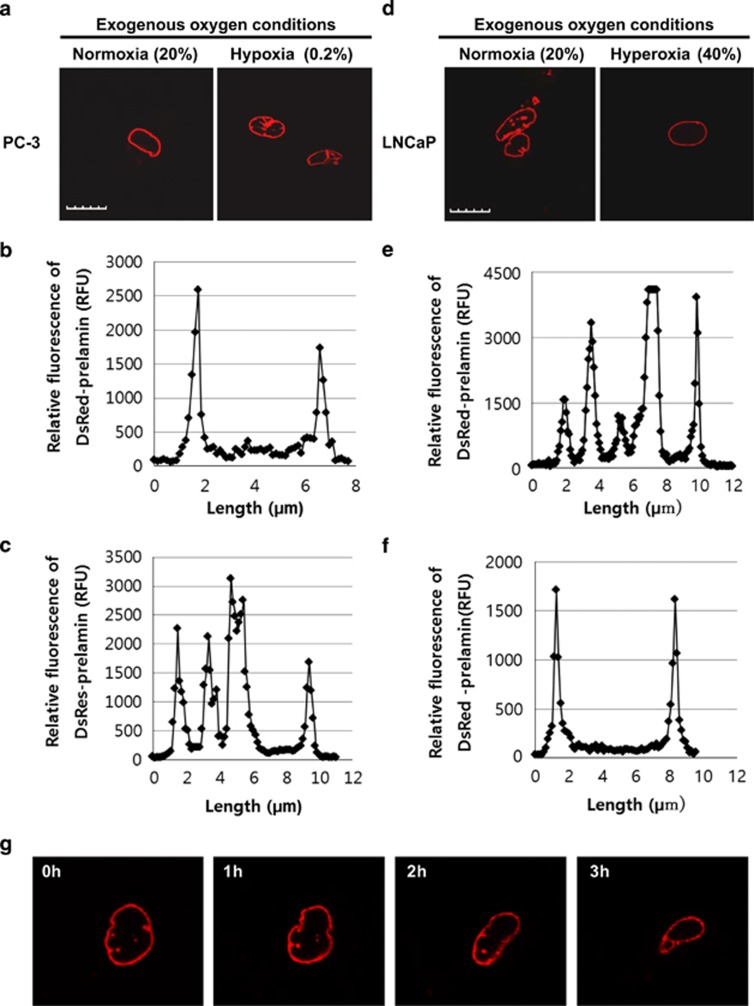
Localization of prelamin is regulated by intracellular oxygen concentration. (**a**) Confocal images of DsRed-prelamin A/C-transfected PC-3 cells. The cells were treated with indicated oxygen conditions with 5% CO_2_. Red fluorescent images show DsRed-prelamin A/C. Scale bar, 10 *μ*m. (**b** and **c**) Each graph illustrates the relative fluorescence values of DsRed-prelamin A/C in PC-3 cells throughout a cell. (**d**) Confocal images of DsRed-prelamin A/C transfected LNCaP cells. The cells were treated with indicated oxygen conditions with 5% CO_2_. Scale bar, 10 *μ*m. (**e** and **f**) Each graph illustrates the relative fluorescence values of DsRed-prelamin A/C in LNCaP cells throughout a cell. (**g**) Time-dependent changes in localization of prelamin in LNCaP at exogenous hyperoxic concentration (40% O_2_/5% CO_2_)

Prenylated proteins often have a consensus sequence, CAAX box, at the C-terminus.^7^ In addition to H-Ras, the current study examined the effect of intracellular oxygen concentration on the membrane localization of other CAAX-box proteins, such as prelamin A/C. Prelamin A/C is a precursor of lamin A and C, which are important nuclear envelope proteins.^15, 16^ The nuclear envelope serves a crucial role in nuclear functions such as cell division and the regulation of transcription and translation.^17^ Defects in nuclear envelope are observed in Hutchinson-Gilford Progeria Syndrome, a genetic disease which has a point mutation in prelamin A leading to form an improper nuclear envelope.^18^ To monitor the localization of prelamin A/C, cells were transfected with DsRed-prelamin A/C.^19^ The majority of prelamin A/C in PC-3 cells was localized to the nuclear membrane, resulting in the appropriate nuclear envelope formation (Figures 4a and b). To examine the effect of changes in oxygen concentration on the nuclear membrane localization of prelamin A/C, DsRed-prelamin A/C-transfected LNCaP and PC-3 cells were incubated in various exogenous oxygen concentrations (Supplementary Figure S2A). In exogenous hypoxia, the formation of the nuclear envelope of PC-3 cells was significantly inhibited (Figure 4a). In addition, prelamin A/C localized inside the nucleus rather than on the nuclear membrane (Figures 4a and c). In contrast, prelamin A/C in LNCaP cells was not only partially localized to the nuclear membrane but also localized inside the nucleus, demonstrating a defect in nuclear envelope formation, similar to that observed in Hutchinson-Gilford Progeria Syndrome (Figures 4d and e).^18^ Exogenous hyperoxic conditions induced prelamin A/C localization to the nuclear membrane and formation of a proper nuclear envelope in LNCaP cells (Figures 4d and f). The time-course video analysis demonstrated a significant reduction in prelamin A/C inside the nucleus and the formation of a proper nuclear membrane within 3 h (Figure 4g and Supplementary Movie 7). These results suggest that prelamin A/C localizes on the nuclear membrane and forms a normal nuclear envelope under high intracellular oxygen concentration. In stark contrast, intracellular hypoxia results in poor prelamin A/C processing, leading to abnormal nuclear envelope formation. Collectively, the precise intracellular localization of CAAX-box proteins, such as H-Ras and prelamin A/C, are regulated by intracellular oxygen concentration.

### Regulation of localization of Rab5a by intracellular oxygen concentration

**Figure 5:**
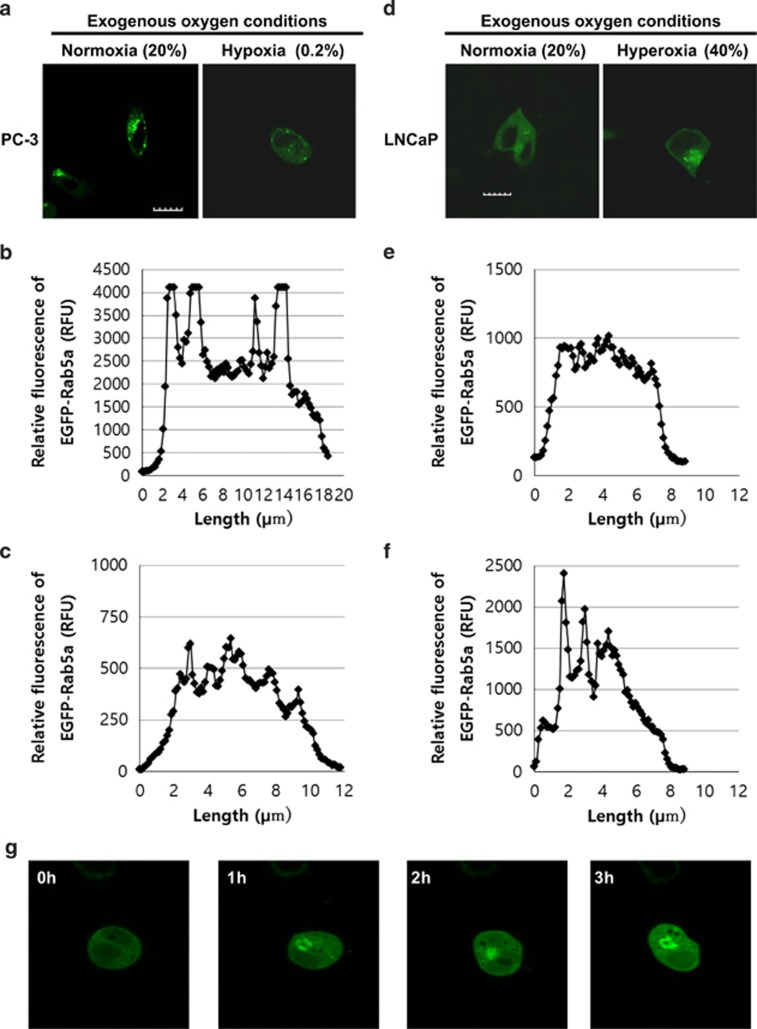
Localization of Rab5a is regulated by intracellular oxygen concentration. (**a**) Confocal images of EGFP-Rab5a transfected PC-3 cells. The cells were treated with indicated oxygen conditions with 5% CO_2_. Green fluorescent images show EGFP-Rab5a. Scale bar, 10 *μ*m. (**b** and **c**) Each graph illustrates the relative fluorescence values of EGFP-Rab5a in PC-3 cells throughout a cell. (**d**) Confocal images of EGFP-Rab5a-transfected LNCaP cells. The cells were treated with indicated oxygen conditions with 5% CO_2_. Scale bar, 10 *μ*m. (**e** and **f**) Each graph illustrates the relative fluorescence values of EGFP-Rab5a in LNCaP cells throughout a cell. (**g**) Time-dependent changes in the localization of EGFP-Rab5a in LNCaP at exogenous hyperoxic concentration (40% O_2/_5% CO_2_)

GGPP is the product from the mevalonate pathway that is transferred to prenylated proteins *via* geranylgeranyltransferases (GGTs).^23^ There are two types of GGTs: GGTI and GGTII (also known as Rab-geranyltransferase). Rab proteins are small GTPases and play an important role in vesicle formation and transport.^21, 22^ Most Rab proteins do not contain the CAAX sequence; instead, they have a single cysteine residue targeted by GGTII.^20^ Rab5a gets geranylgeranylated before being transported to the membrane.^23^ We transiently transfected cells with pEGFP-Rab5a, and monitored the localization of the protein (Supplementary Figure S2B). In endogenously normoxic PC-3 cells, Rab5a was observed mostly in endomembrane organelles (Figures 5a and b). These endomembrane organelles are the early endosomes, shown in other studies done by Ali *et al.*^11^ Next, the effect of oxygen concentration on the localization of Rab5a in PC-3 and LNCaP cells was examined (Supplementary Figure S2B). In contrast to the control, exogenous hypoxia induced Rab5a in PC-3 cells to be in the cytosol, indicating less localization in the early endosomes (Figures 5a and c). In contrast, Rab5a in LNCaP cells displayed a diffused staining pattern throughout the cells indicating less localization in the early endosomes but more in the cytosol (Figures 5d and e). Rab5a was localized more in the early endosomes in the exogenously hyperoxic conditions (Figures 5d and f). The time-course video analysis of LNCaP cells in exogenous hyperoxia revealed a visible increase in Rab5a membrane localization in the early endosomes very rapidly (Figure 5g), even within 30 min (Supplementary Movie 8). These results indicate that the localization of prenylated proteins without CAAX sequences is also regulated by intracellular oxygen concentration. Rab proteins are greatly involved in vesicle transport as they regulate the fusion of vesicles to target membranes. Intracellular oxygen concentration may be able to regulate membrane localization of Rab proteins leading to control vesicle transport events.

### Determination of the role of intracellular oxygen concentration controlled by mitochondrial respiration in prenylated proteins

**Figure 6:**
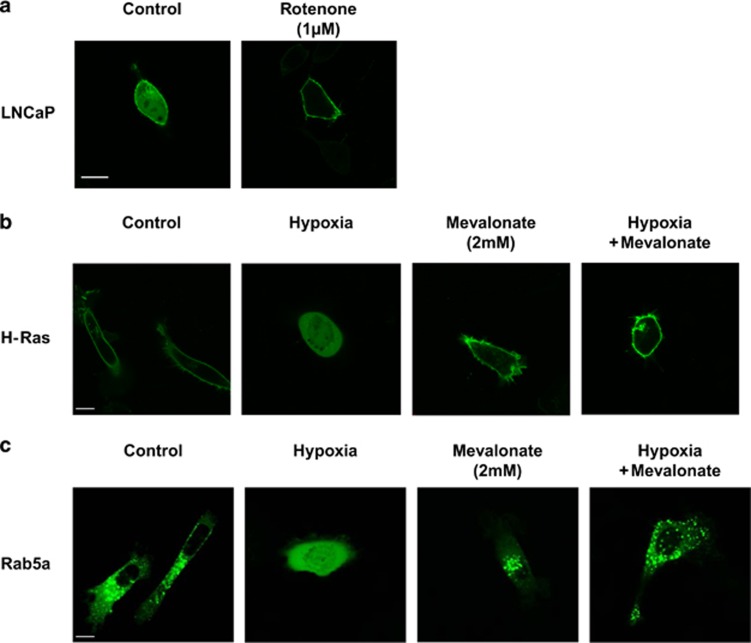
Intracellular oxygen concentration is a key molecule regulating the localization of the prenylated proteins. (**a**) Confocal images of EGFP-H-Ras-transfected LNCaP cells. The cells were treated with or without 1 *μ*M of Rotenone for 6 h. Scale bar, 10 *μ*m. (**b** and **c**) Confocal images of EGFP-H-Ras- and EGFP-Rab5a-transfected PC-3 cells, respectively. Scale bar, 10 *μ*m. EGFP-H-Ras- or EGFP-Rab5a-transfected PC-3 cells were treated with 2 mM mevalonate and/or 0.2% O_2_/5%CO_2_

Hypoxia stimulates the degradation of HMGR in the mevalonate pathway. The mevalonate pathway produces FPP and GGPP to induce prenylation. We investigated whether the oxygen, determined by mitochondrial respiration, is the critical molecule to regulate localization (prenylation) *via* HMGR. Our previous studies have shown that Rotenone, a mitochondrial respiratory inhibitor, increased intracellular oxygen concentration^4, 24^ and increased HMGR expression and Ras activation.^4^ In the current report, Rotenone drastically induced the membrane localization of H-Ras, indicating that the mitochondrial respiratory activity controlled the oxygen concentration and regulated membrane localization of H-Ras (Figure 6a). pEGFP-H-Ras- and pEGFP-Rab5a transfected-PC-3 cells were incubated in the exogenous hypoxia with or without mevalonate, the product of HMGR. As described above, hypoxic conditions inhibited the membrane localization of EGFP-H-Ras in the PC-3 cells, whereas, mevalonate enhanced its membrane localization slightly (Figure 6b). When the cells were treated with mevalonate and incubated in hypoxic conditions for 6 h, H-Ras was localized in the Golgi and the plasma membrane, indicating that the inhibition of H-Ras membrane localization by the exogenous hypoxia was completely reversed by mevalonate (Figure 6b). Similarly, EGFP-Rab5a was predominantly in the cytosol, exhibiting a diffused staining pattern, after the exogenous hypoxia treatment (Figure 6c). Mevalonate increased the localization in the early endosomes. The combination of the exogenous hypoxic conditions and mevalonate treatment restored the localization of Rab5a to the early endosomes (Figure 6c). The results suggest that intracellular oxygen is the key molecule regulating HMGR by which the isoprenoids for prenylation was produced in the mevalonate pathway.

## Discussion

Here, we showed that the intracellular oxygen concentration regulated membrane localization and activation of various prenylated proteins. The regulation of the prenylation is through the hypoxia-stimulated degradation of HMGR in the mevalonate pathway. The exogenous oxygen conditions were able to manipulate intracellular oxygen concentration and the localization of the CAAX-box-containing proteins, H-Ras and prelamin A/C, and non-CAAX-box protein, Rab5a.

Reduction of mitochondrial respiration increases intracellular oxygen concentration.^4^ Several mechanisms can inhibit oxygen consumption, such as changes of mtDNA^25^ and mutations to specific nuclear genes.^26, 27^ Another study implies that changes in expression of glycolytic genes can lead to the reduction of oxidative phosphorylation and an increase in glycolysis.^28^

**Figure 7:**
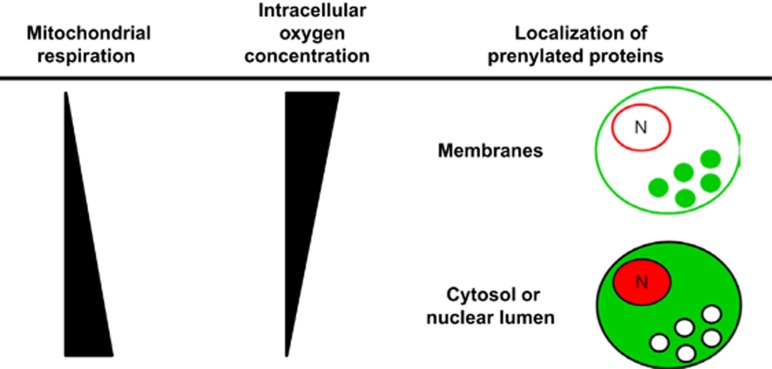
Schematic diagram illustrating that intracellular oxygen concentration, determined by oxidative phosphorylation, regulates prenylation. Green color represents H-Ras and Rab5a and red color represents prelamin A/C

We showed here a direct link between the reduction of mitochondrial respiration and the membrane localization of prenylated proteins. The reduction of oxidative phosphorylation increases intracellular oxygen concentrations, leading to the prenylation and membrane localization of the proteins (Figure 7).

Our data showed that intracellular oxygen concentration regulated the membrane localization and activation of Ras, suggesting that the Warburg effect was the cause for Ras activation. Several studies indicate that Ras is greatly involved in the reprogramming of metabolism of cancer.^5^ Ras plays an important role in an increase of glycolysis by inducing glucose uptake *via* GLUT1 and increasing transcription and translation of glycolytic enzymes,^5^ and is considered the cause for the Warburg effect. In addition, a recent study reported that K-Ras transformation(G12V) led to mitochondrial dysfunction and a metabolic switch from oxidative phosphorylation to glycolysis.^29^ Reduction of oxidative phosphorylation can lead to membrane localization and subsequent activation of Ras. The activated Ras then triggers the reprogramming of metabolism of cancer cells to increase glycolysis, which further amplifies the activation of Ras. The Warburg effect and the activation of Ras may generate positive feedback.

Prelamin A/C and Rab5a are essential proteins which are prenylated to localize at the final places. Mutations on the farnesylated CaaX site of prelamin A/C are indicated to cause HGPS. Here we discovered that hypoxia impaired the formation of nuclear envelope and led prelamin A/C to localize inside of the nuclear lumen, mimicking the nuclear envelope in the HGPS patients.^30^ We also observed that early endosome localization of Rab5a was inhibited by hypoxia, and the hyperoxic conditions or mevalonate were able to reverse the inhibitory effect of the intracellular hypoxia. The detailed mechanism needs to be elucidated.

Prenylation is an important posttranslational modification for biological functions. There are more than 300 CAAX-box proteins in a cell and they are essential proteins such as G-proteins, helicases, phosphatases, kinases, phospholipases, etc.^31^ Small GTP-binding proteins control many other important intracellular processes, including trafficking of membrane compartments within eukaryotic cells and cytoskeleton function.^32^ Prelamin and helicases regulate gene transcription and translation.^18^ Prenylated proteins, such as Ras, play a central role for cancer development.^5^ Reduction of mitochondrial respiration enhances cancer to exhibit more aggressive phenotypes^4^ suggesting that endogenous normoxia-induced prenylation may induce a more aggressive phenotype of cancer. Therefore, the prenylated proteins could be targeted for anti-cancer drug development. Epidemiological analysis showed that in statin users, the number of cancer-related deaths is reduced. Inhibitors of prenyltransferase (PTI) have been subjected in preclinical and clinical studies for its efficacy as cancer treatment.^33, 34^ Regulation of intracellular oxygen concentration may enhance the efficacy of PTIs as intracellular oxygen concentration can regulate induction of FPP and GGPP *via* HMGR. Translocation of H-Ras and Rab5a by enhancing intracellular oxygen was quite rapid (30 min) but that of prelamin A/C was slow (3 h). This could be that the prenylated prelamin requires more time to localize to the nucleus and then translocate to the nuclear membrane.

Hypoxia-stimulated proteasomal degradation of HMGR is mediated by the accumulation of sterols in the mevalonate pathway.^10^ The final product of the mevalonate pathway is cholesterol, and enzymes catalyzing cholesterol from lanosterol are oxygen-requiring enzymes. During hypoxia, the oxygen-requiring enzymes are inhibited; therefore, cholesterol intermediate molecules are accumulated in a cell. The accumulation of the sterols triggers insig-mediated ubiquitination of HMGR, leading to the degradation of the protein.^35^ Direct measurement of amount for lanosterol and its derivative molecules in a cell will be necessary to determine the role of intracellular oxygen concentration in the expression of HMGR.^35^

Several reports indicate that HMGR is transcriptionally upregulated. In HepG2 cells, weak hypoxia led to an increase in transcription of HMGR.^36^ The effect of hypoxia in murine models has been investigated using ischemic/hypoxic conditions, and it increased HMGR expression transcriptionally.^37^ In contrast, hypoxia affected only lipid metabolism without affecting cholesterol synthesis.^38^ Weak hypoxia (2% O_2_) may enhance the mRNA level of HMGR though activation of SREBP by HIF-1. Our data showed that low intracellular oxygen concentration induced the posttranscriptional modification (i.e., degradation of the protein *via* ubiquitination), possibly through Insig activation by accumulation of sterol intermediates.^10^ Therefore, it is very likely that weak hypoxia may induce transcriptional activation of HMGR and strong hypoxia (0.2% O_2_) will induce proteolytic degradation of HMGR.

The current report investigated the roles of intracellular oxygen concentration in the activation of prenylated proteins. Many prenylated proteins were involved in numerous essential pathways for cell growth and death, differentiation, and proliferation. The main focus of this paper is to elucidate the link between oxygen changes and the activation of Ras and other prenylated proteins, and it will provide better insight to progression of the many human diseases, such as cancer and aging.

## Materials and Methods

### Materials

Lovastatin, mevalonate, and sodium sulfide were purchased from Sigma-Aldrich (St. Louis, MO, USA).

### Cell culture and transfection

PC-3 was purchased from ATCC (Manassa, VA, USA) and LNCaP was purchased from UROCOR (Minnetonka, NM, USA). LNCaP and PC-3 were maintained in RPMI (Life Technologies, Grand Island, NJ, USA) plus 5% fetal calf serum (Life Technologies). All cell lines were maintained at 37 °C with room air plus 5% CO_2_ unless noted for specific oxygen experiments. PC-3 and LNCaP cells were plated and grown in glass-bottom 35 mm dish to be approximately 90% confluency then transfected with EGFP-H-Ras,^10^ EGFP-Rab5a,^10^ or DsRed-Lamin.^19^ The transiently transfected cells were subjected to confocal microscopy.

### Laser confocal microscopy

Cells were maintained in a stage-mounted atmospheric box (Pathology Devices, Westminster, MD, USA) at 37 °C, 5% CO_2_, and 75% humidity during the course of the experiments. All samples were analyzed on an Olympus Fluoview FV1000 laser confocal microscope (Olympus, Center Valley, PA, USA). For detection of intracellular oxygen concentration, acetylacetonatobis [2-(2′-benzothienyl)pyridinato-kN,kC3']iridium(III) (BTP) (515 excitation/620 emission) was utilized in the indicated experiments at a concentration of 5 *μ*M in RPMI medium. BTP has been described in detail by Zhang *et al.*^13^ BTP is a phosphorescent compound that is phosphorescent in low-oxygen conditions and is quenched in the presence of oxygen. The extent of quenching is dependent upon intracellular oxygen concentration. Samples were incubated in the presence of BTP for 1 h before imaging. In all experiments, cells were plated and grown overnight at a cell density of 1 × 10^5^ cells in 35 mm dishes with inset cover slips (Mattek, Ashland, MA, USA). For hypoxic experiments, cells were incubated in the atmosphere box for 6 h at 0.2% O_2_ following addition of BTP. For hyperoxic experiments, cells were incubated in the atmosphere box for 6 h at 40% O_2_ following addition of BTP. For 20% O_2_ experiments, cells were incubated in the presence of BTP in a normal cell culture incubator for 1 h. For the transiently transfected cells, they were placed in the atmospheric box at the various oxygen concentrations as indicated in the figures. Images from all microscopy experiments were processed using the FV10-ASW 3.1 Viewer (Olympus).

### Determination of oxygen concentration surrounding cells using oxoplate

Oxoplate OP96F plates were purchased from PreSens (Innovative Instruments, Indian Trail, NC, USA). The Oxoplate system has been described in detail by Cook *et al.*^4^ LNCaP and PC-3 were grown in 10 cm culture dishes under normal culture conditions until the cells had reach approximately 80% confluency and then were trypsinized to obtain cell suspensions. Cells were added to the Oxoplate at a concentration of 1 × 10^6^ cells/ml in RPMI plus 5% fetal calf serum (200 *μ*l final volume). Fluorescence in each well was then measured every 5 min for 3 h at 37 °C in a plate reader (Synergy HT, BIO-TEK, Winooski, VT, USA). The oxygen concentration in the wells at each time point was calculated. All samples were run in triplicate and the indicated error bars were standard error of each sample.

### Western blotting analysis

Cells were transiently transfected with EGFP-H-Ras plasmid and then lysed in radioimmunoprecipitation assay buffer added with DTT (0.5 mM), and protease and phosphatase inhibitors (100 × Halt protease and phosphate inhibitor cocktail, no. 1861281, Thermo Scientific, Waltham, MA, USA). The protein concentration of the cell lysates was determined by a protein assay (Bio-Rad Protein Assay, Bio-Rad, Hercules, CA, USA). The cell were subjected to SDS-PAGE, and then transferred to immobilon-P membrane (Millipore, Billerica, MA, USA). After blocking with 1.5% bovine serum albumin in TBS (blocking solution), the membrane was incubated overnight (4 °C) in primary antibody diluted (1 : 1000) in 1.5% bovine serum albumin (Sigma-Aldrich). Antibodies utilized in the current study are as follows: rabbit anti-phospho ERK(T202/Y204) (no. 9101), rabbit anti-total ERK (no. 9102), rabbit anti-HMGR (Millipore ABS#229), and mouse anti-GAPDH (no. MAB374, Millipore). The membrane was then washed in TBS, and incubated (1 h at RT) with HRP-linked anti-mouse IgG or anti-rabbit IgG diluted (1 : 5000) in 1.5% bovine serum albumin-TBS (no. 7074 and no. 7076, respectively, Cell Signaling, Beverly, MA, USA). The membrane was then washed in TBS, and analyzed with ECL Plus (GE Healthcare Bio-Sciences AB, Uppsala, Sweden) by autoradiography film.

## Abbreviations

mtDNA: mitochondrial DNA
PCa: prostate cancer
HMGR: 3-hydroxy-3-methyl-glutaryl-coenzyme A reductase
FPP: farnesylpyrophosphate
GGPP: geranylgeranylpyrophosphate
HMG-CoA: 3-hydroxy-3-methyl-glutaryl-CoA
BTP: acetylacetonatobis [2-(2'-benzothienyl)pyridinato-kN,kC3']iridium(III)
GGT: geranylgeranyltransferases
PTI: prenyltransferase inhibitors
