# Supplementary material for: Intracellular oxygen determined by respiration regulates localization of Ras and prenylated proteins
Source: Cell Death Dis. 2015 Jul 16;6(7):e1825–. doi: 10.1038/cddis.2015.64 (PMC4650746; doi:10.1038/cddis.2015.64)
Supplement: Supplementary Information [file cddis201564x1.doc]

**Supplementary Figure 1. Endogenous oxygen concentration regulated localization of H-Ras.** (A) Schematic diagrams of the localization of EGFP-H-Ras. LNCaP exhibits endogenous hypoxia (Fig. 1E, top-left panel) therefore, prenylation and membrane localization (to the plasma membrane and Golgi apparatus) of EGFP-H-Ras are prevented (indicated by green color of cytosol). In exogenous hyperoxia, intracellular oxygen concentration increases (Fig. 1E, top-right panel) and the prenylation and membrane localization of EGFP-H-Ras are increased (indicated by green color of plasma membrane and Golgi apparatus). PC-3 exhibits endogenous normoxia (Fig. 1E, bottom-left panel), and EGFP-H-Ras is localized on the membranes (the plasma membrane and Golgi apparatus). In exogenous hypoxic condition, intracellular oxygen concentration decreases (Fig. 1E, bottom-right panel) and the prenylation and membrane localization of EGFP-H-Ras are inhibited (indicated by green color of cytosol). (B and C) Representative figures of a method for computing the relative fluorescence value of the membrane localization of H-Ras. A line (B) was drawn across a cell and the histogram (C) of the relative fluorescence values along the line was obtained. The green rectangles (b1 and b2) in the histogram enclose the relative fluorescence values measured at the plasma membrane. The black rectangle (a) in the histogram encloses the relative fluorescence values measured within the cytosol. The computation of the ratio of localization of H-Ras in membrane to cytosol per cell was performed using following equation:


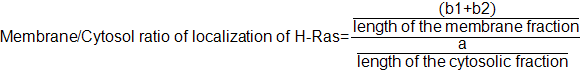


**Supplementary Figure 2. Endogenous oxygen concentration regulated localization of prelamin A/C and Rab5a.** (A) Schematic diagrams of the localization of prelamin A/C. LNCaP exhibits endogenous hypoxia (Fig. 1E, top-left panel), and membrane localization of DsRed-prelamin A/C (to the nucleus) is prevented (indicated by the red color filled in nucleus). In exogenous hyperoxic conditions, intracellular oxygen concentration increases (Fig. 1E, top-right panel) and the prenylation and the membrane localization of prelamin A/C are increased (indicated by red color of nuclear membrane). PC-3 exhibits endogenous normoxia (Fig. 1E, bottom-left panel), and prelamin A/C is localized on the nuclear membranes. In exogenous hypoxic conditions, intracellular oxygen concentration decreases (Fig. 1E, bottom-right panel) and the prenylation and membrane localization of prelamin A/C are inhibited. (B) Schematic diagrams of the localization of Rab5a. LNCaP exhibits endogenous hypoxia (Fig. 1E, top-left panel), and membrane localization of Rab5a (to the early endosomes) is prevented (indicated by the green color of cytosol). In exogenous hyperoxic conditions, intracellular oxygen concentration increases (Fig. 1E, top-right panel) and the prenylation and the membrane localizations of Rab5a are increased (indicated by the green color of the early endosomes). PC-3 exhibits endogenous normoxia (Fig. 1E, bottom-left panel), and Rab5a is localized on the early endosomes. In exogenous hypoxic conditions, intracellular oxygen concentration decreases (Fig. 1E, bottom-right panel) and the prenylation and membrane localization of Rab5a are inhibited.

**Supplementary Movies**

**Supplementary Movie 1. Endogenous oxygen regulates localization of H-Ras in LNCaP.** 3-dimensional (3D) video of z-stack data from EGFP-H-Ras transfected LNCaP cells in normoxic conditions. LNCaP cells were cultured at 20% O_2_/5% CO_2_.

**Supplementary Movie 2. Endogenous oxygen regulates localization of H-Ras in PC-3.** 3-dimensional (3D) video of z-stack data from EGFP-H-Ras transfected PC-3 cells in normoxic conditions. PC-3 cells were cultured at 20% O_2_/5% CO_2_.

**Supplementary Movie 3. Lovastatin, HMGR inhibitor, inhibits prenylation and membrane localization of EGFP-H-Ras in PC-3.** 3-dimensional (3D) video of z-stack data from EGFP-H-Ras transfected PC-3 cells treated with lovastatin (5μM) in normoxic conditions. PC-3 cells were cultured at 20% O_2_/5% CO_2_.

**Supplementary Movie 4. Intracellular oxygen concentration regulates membrane localization of H-Ras.** 3-dimensional (3D) video of z-stack data from EGFP-H-Ras transfected PC-3 cells in hypoxic conditions. PC-3 cells were cultured at 0.2% O_2_/5% CO_2_ for 6 hours.

**Supplementary Movie 5. Intracellular oxygen concentration regulates membrane localization of H-Ras.** 3-dimensional (3D) video of z-stack data from EGFP-H-Ras transfected LNCaP cells in hyperoxic conditions. LNCaP cells were cultured at 40% O_2_/5% CO_2_ for 6 hours.

**Supplementary Movie 6. Exogenous hyperoxic conditions enhance membrane localization of H-Ras in LNCaP (time-course video).** Exogenous hyperoxic conditions enhance membrane localization of H-Ras in LNCaP (time-course video) EGFP-H-Ras transfected LNCaP cells were incubated in exogenous hyperoxic conditions (40% O_2_) for 6 hours. Images were collected every 10min.

**Supplementary Movie 7. Endogenous oxygen concentration regulates localization of prelamin A/C**. Exogenous hyperoxic conditions enhance membrane localization of prelamin A/C in LNCaP (time-course video). DsRed-prelamin A/C transfected LNCaP cells were incubated in exogenous hyperoxic conditions (40% O_2_) for 6 hours. Images were collected every 10min.

**Supplementary Movie 8. Endogenous oxygen concentration regulates localization of Rab5a**. Exogenous hyperoxic conditions enhance membrane localization of Rab5a in LNCaP (time-course video). EGFP-Rab5a transfected LNCaP cells were incubated in exogenous hyperoxic conditions (40% O_2_) for 6 hours. Images were collected every 10min.
