# Supplementary figures and images for: Intracellular oxygen determined by respiration regulates localization of Ras and prenylated proteins
Source: Cell Death Dis. 2015 Jul 16;6(7):e1825–. doi: 10.1038/cddis.2015.64 (PMC4650746; doi:10.1038/cddis.2015.64)

A

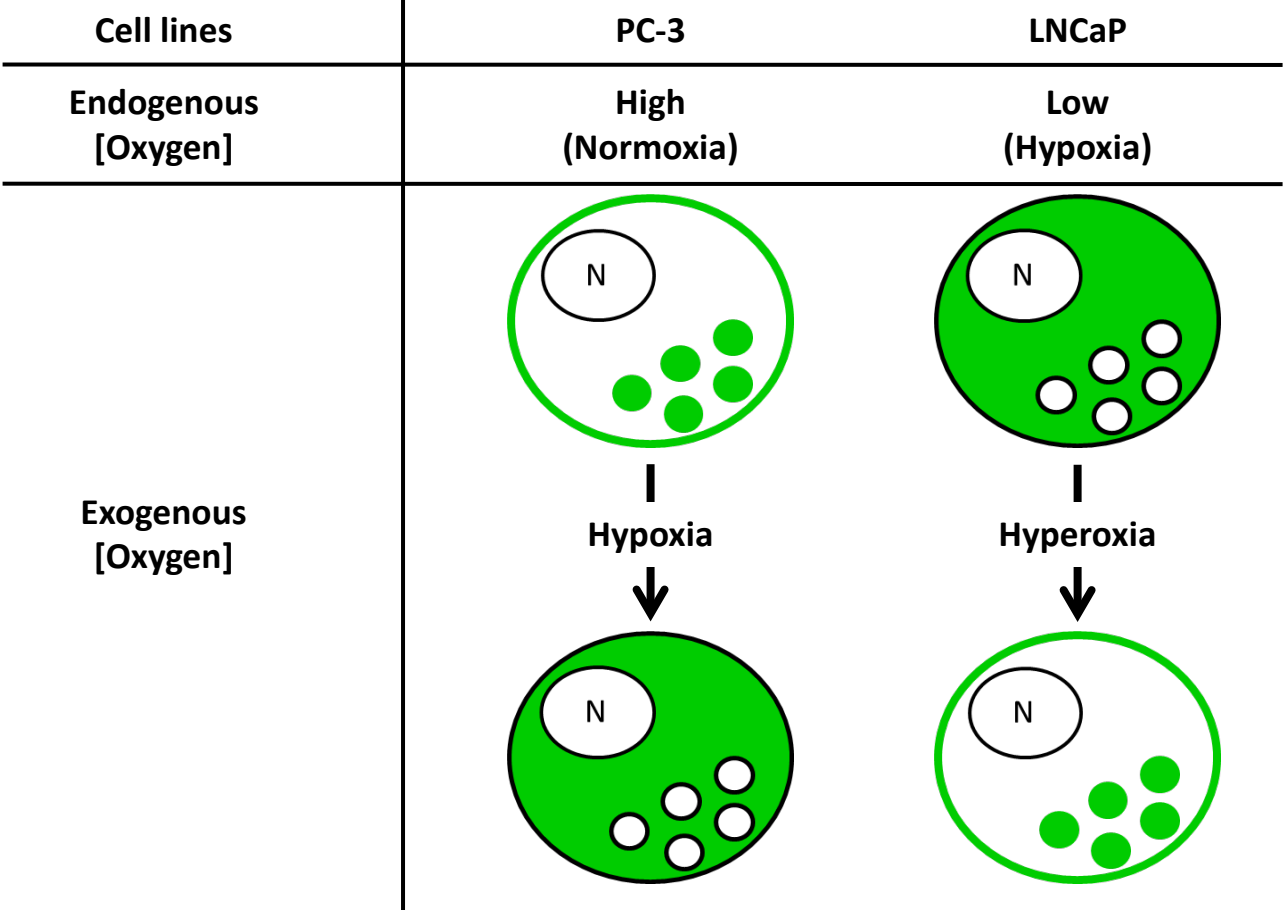

B

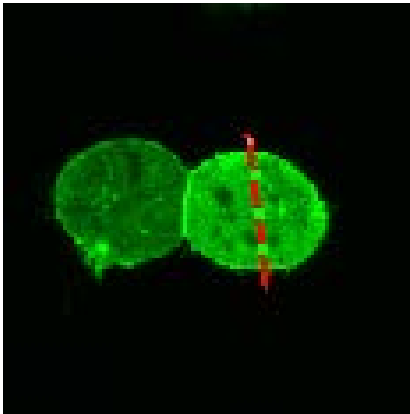

C

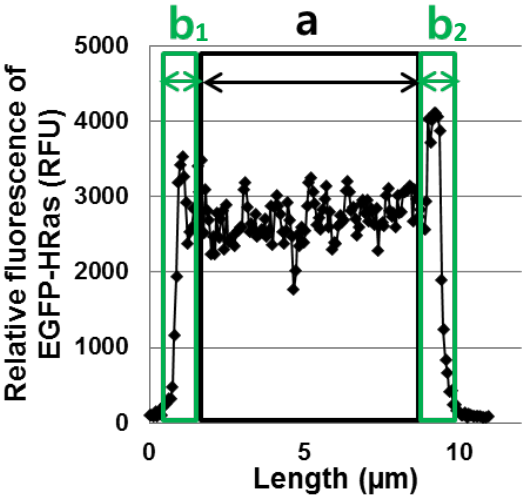

Supplement: Supplementary Figure S1 [file cddis201564x2.pdf]

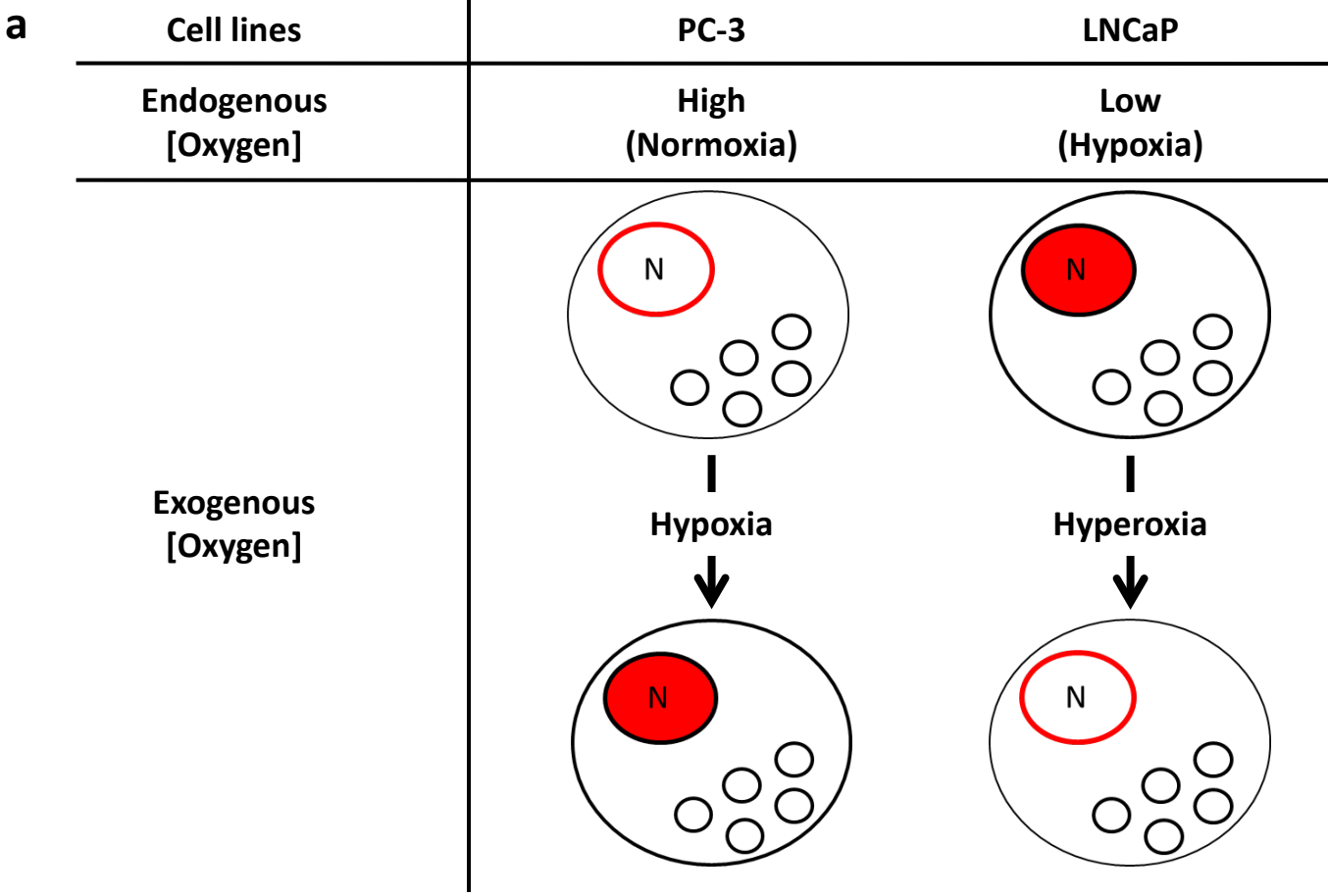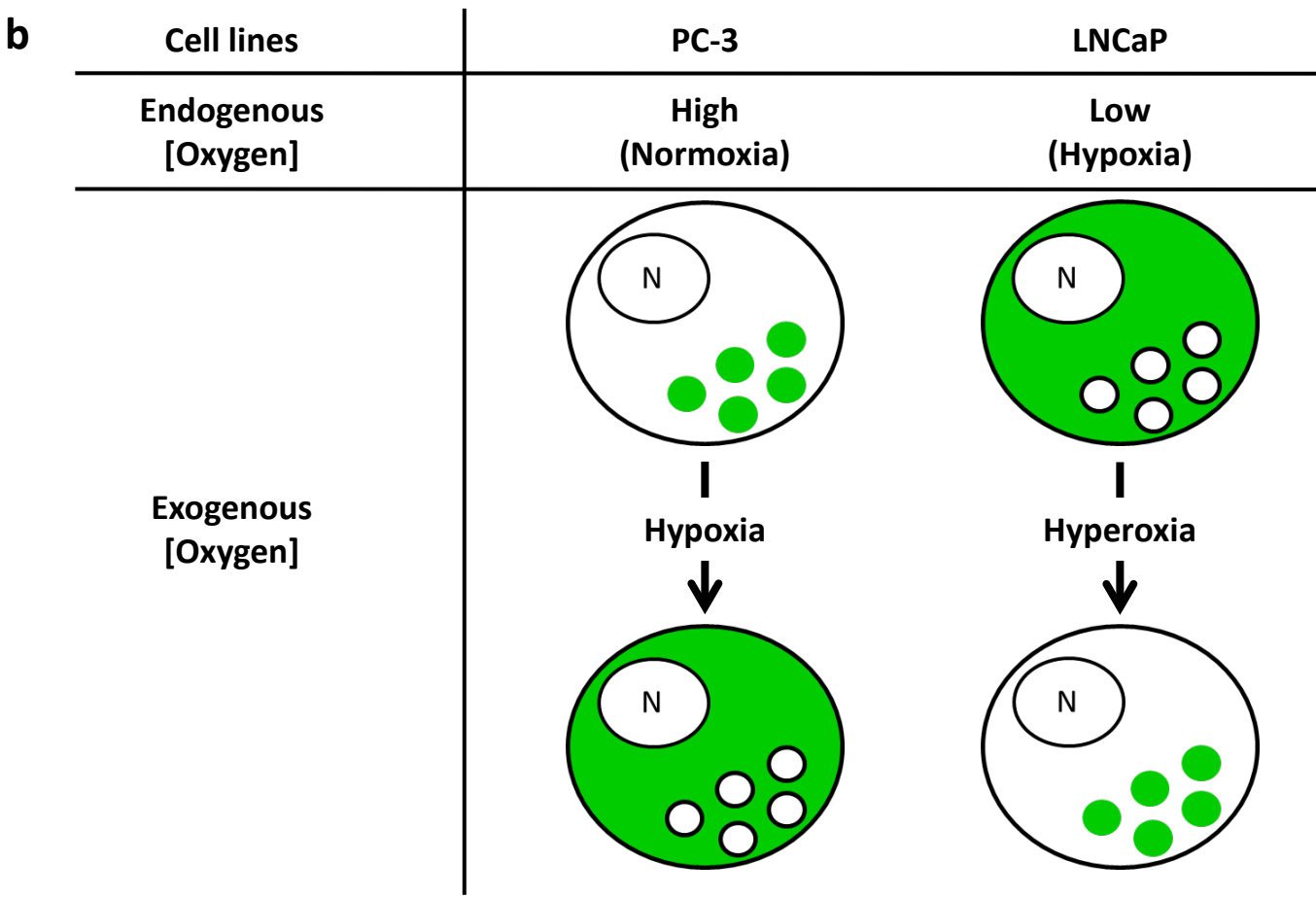

Supplement: Supplementary Figure S2 [file cddis201564x3.pdf]
